# Characterization of the whole plastome of *Dipentodon Sinicus* (Dipentodontaceae)

**DOI:** 10.1080/23802359.2019.1667914

**Published:** 2019-09-20

**Authors:** Changkun Liu, Lili Liu, Lei Jin, Zhenyan Yang, Yunheng Ji

**Affiliations:** a Yunnan Key Laboratory for Integrative Conservation of Plant Species with Extremely Small Population, Kunming Institute of Botany, Chinese Academy of Sciences, Kunming, Yunnan, China;; b Key Laboratory for Plant Diversity and Biogeography of East Asia, Kunming Institute of Botany, Chinese Academy of Sciences, Kunming, Yunnan, China;; c College Pharmacy, Chengdu University of TCM, Chengdu, Sichuan, China;; d College of Traditional Chinese Medicine, Guangdong Pharmaceutical University, Guangzhou, Guangdong, China

**Keywords:** *Dipentodon sinicus*, Dipentodontaceae, Huerteales, plastome

## Abstract

In this study, we sequenced and assembled the whole plastome of *Dipentodon sinicus*. The plastome was 158,020 bp in length and consisted of a pair of inverted repeat regions (IRs, 26,551 bp), a large single-copy region (LSC, 86,624 bp) and a small single-copy region (SSC, 18,294 bp). It encoded 114 unique genes (80 protein-coding genes, 30 tRNA genes and 4 rRNA genes). Phylogenetic analysis showed that *Dipentodon sinicus* was sister to *Tapiscia sinensis*.

Dipentodontaceae Merrill is a small family in the malvids (Angiosperm Phylogeny Group 2016), which only includes two genera, namely, *Dipentodon* Dunn and *Perrottetia* Kunth (Ma and Bartholomew 2008). However, its phylogenetic position has long been contentious. Dipentodontaceae was placed in Rosales between Hamamelidaceae and Rosaceae by Merrill (1941) when it established. Hutchinson (1959) then transferred the family into Olacales based on the free basal placentation. Cronquist (1981) included the family Dipentodonaceae in Santalales, while Takhtajan (1997) defined it as a member of Violales. Recently, establishing the order Huerteales to accommodate the family was supported by molecular evidence (Angiosperm Phylogeny Group 2009; Worberg et al. 2009; Angiosperm Phylogeny Group 2016).

Although lots of studies have been performed, available genomic resources of Dipentodontaceae are still limited, which severely hinders our understanding for the evolution of the family. Here, we presented the whole plastome of *Dipentodon sinicus* Dunn, the only member of the genus *Dipentodon*. Samples of *Dipentodon sinicus* were collected from Gongshan county (27°44′49′′N, 98°40′20′′E), Yunnan province, China. Specimen (jyh33135) was deposited in the Herbarium of Kunming Institute of Botany, Chinese Academy of Sciences (KUN). Total DNA was extracted from the silica-gel-dried leaf using the modified CTAB method (Yang et al. 2014). Ensuing, purified DNA was fragmented into ∼500 bp to construct paired-end library. We then prepared Illumina librariy according to the manufacturer’s protocol. The library was sequenced on the Illumina HiSeq 2000 system. Assembly for the whole plastome of *Dipentodon sinicus* was performed using the method described by Jin et al. (2018) with the plastome of *Tapiscia sinensis* (MF926267) as the reference. We then annotated the plastome in Geneious 10.2 (Kearse et al. 2012), and manually checked for start and stop codons and intron/exon boundaries. Validated complete plastome of *Dipentodon sinicus* was deposited in GenBank under accession number MN275517.

Overall size of the *Dipentodon sinicus* plastome was 158,020 bp, and it presented a typical quadripartite structure, including a pair of inverted repeat regions (IRs, 26,551 bp) divided by a large single-copy region (LSC, 86,624 bp) and a small single-copy region (SSC, 18,294 bp). The plastome of *Dipentodon sinicus* possessed 114 unique genes, including 80 protein-coding genes, 30 tRNA genes, and four rRNA genes. Among them, twelve protein-coding genes (*atpF,* *ndhA,* *ndhB,* *petB,* *petD,* *rpl16,* *rpl2,* *rpoC1,* *rps12,* *rps16,* *clpP* and *ycf3*) and six tRNAs (*trn*A-UGC, *trn*G-UCC, *trn*I-GAU, *trn*K-UUU, *trn*L-UAA and *trn*V-UAC) contained at least one intron.

To investigate the phylogeny of *Dipentodon sinicus*, we reconstructed the maximum likelihood tree based on twenty whole plastomes using the software RAxML version 8.2.11 (Stamatakis 2014) with 1,000 bootstrap replicates. *Larrea tridentata* was used as outgroup. The result strongly supported that *Dipentodon sinicus* was sister to *Tapiscia sinensis* (Figure 1).

**Figure 1.**
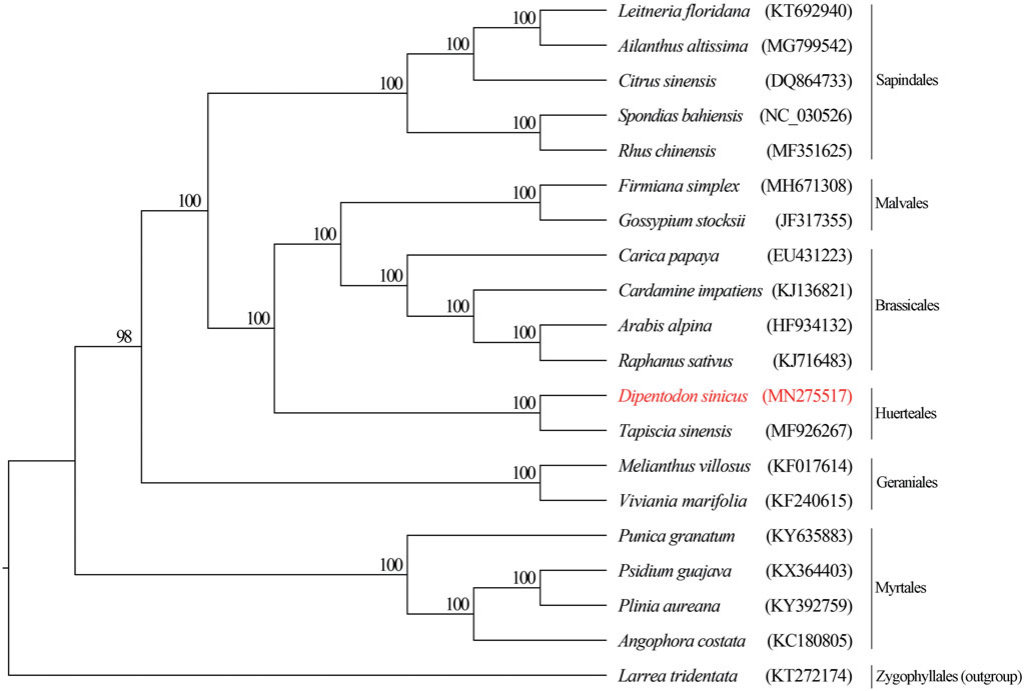
Maximum likelihood (ML) tree was generated with whole plastomes. The bootstrap values are presented above the clade.
